# The pattern and trend of COVID-19 spread in the Eastern Mediterranean Region: curve-fitting models estimation

**DOI:** 10.11604/pamj.supp.2020.35.2.23269

**Published:** 2020-06-08

**Authors:** Mohannad Abed Alfattah Al Nsour, Yousef Saleh Khader, Haitham Majed Nazzal

**Affiliations:** 1 Global Health Development/Eastern Mediterranean Public Health Network, Jordan; 2 Department of Public Health, Jordan University of Science and Technology, Jordan

**Keywords:** COVID-19, pattern, Eastern Mediterranean Region

## Abstract

**Introduction:**

This study aimed to use the Curve Estimation Procedure to assess the pattern and trend of COVID-19 spread in the countries of the Eastern Mediterranean Region (EMR) by finding the model best fit for the observed COVID-19 data in each country between 20 February 2020-21 April 2020.

**Methods:**

The number of daily confirmed COVID-19 cases, recovered cases, and during the period 20 February 2020-21 April 2020 in 21 countries in EMR were extracted from the WHO situation reports. The Curve Estimation procedure was used to produce different curve estimation regression models for the observed data in each country.

**Results:**

During this observed period, the total number of confirmed cases, recovered cases, and deaths in the region were 138673, 71343, and 6291, respectively. The overall fatality rate in the region was 4.5%. The quadratic model and the cubic model follows the observed data points fairly well during the observed time period in five and nine countries, respectively. The exponential model (Y = b0 * (e**(b1 * t))), the growth model (Y = e**(b0 + (b1 * t))), and the compound model (Y = b0 * (b1**t)) were the best fit for data during the observed time period in two, three, and two countries, respectively.

**Conclusion:**

The pattern of COVID-19 spread differed between countries in the EMR. This might reflect the variations in testing and implementation of public health measures. The best curve-fitting model was demonstrated for each country and it can be used for very short-term predictions.

## Introduction

As of 25th April 2020, 159157 people are confirmed to be infected in The Eastern Mediterranean Region (EMR). While around 82160 cases are already recovered in the EMR, the death toll reached to over 152,328 [1]. Epidemiological models during outbreaks are very helpful for policy makers and public health professionals to make key decisions about the appropriate public health measures and interventions. During COVID-19, many models were developed to help in making decisions on social distancing measures, testing and contact tracing, training of health professionals, resources needed, utilization of resources within a health system, and measures that should be taken to reduce COVID-19´s impact. Depending on the purpose of epidemiological models, they can be used to describe the current situation, predict what could happen in the next week or month, and assess the effectiveness of preventive measures. Curve fitting, also known as regression analysis, is used to find the “best fit” line or curve for a series of data points. It is the process of specifying the model that provides the best fit to the specific curves in the dataset and it can be used as an aid for data visualization [2,3]. This study aimed to use the Curve Estimation Procedure to assess the pattern and trend of COVID-19 spread in the countries of the EMR by finding the model that best fit the observed COVID-19 data in each country between 20 February 2020-21 April 2020.

## Methods

The number of daily confirmed COVID-19 cases, recovered cases, and deaths during the period 20 February 2020-21 April 2020 in Eastern Mediterranean countries were extracted from the World Health Organization situation reports [4]. All countries of the EMR are included except Yemen because only one case was reported during the observed period. Data were entered in the IBM SPSS version 24 (IBM Corp. Released 2016. IBM SPSS Statistics for Windows, Version 24.0. Armonk, NY: IBM Corp.) The Curve Estimation procedure was used to produce different curve estimation regression models for the observed data in each country. In all models, the dependent variable (Y) was the number of confirmed cases and the independent variable was time (t). A total of 11 regression models were tested and compared to find the model that best fits the COVID-19 data during the observed period for each country: 1) Linear. Model whose equation is Y = b0 + (b1 * t). The daily confirmed COVID-19 cases were modeled as a linear function of time. 2) Logarithmic. Model whose equation is Y = b0 + (b1 * ln(t)). 3) Inverse. Model whose equation is Y = b0 + (b1 / t). 4) Quadratic. Model whose equation is Y = b0 + (b1 * t) + (b2 * t**2). 5) Cubic. Model that is defined by the equation Y = b0 + (b1 * t) + (b2 * t**2) + (b3 * t**3). 6) Power. Model whose equation is Y = b0 * (t**b1) or ln(Y) = ln(b0) + (b1 * ln(t)). 7) Compound. Model whose equation is Y = b0 * (b1**t) or ln(Y) = ln(b0) + (ln(b1) * t). 8) S-curve. Model whose equation is Y = e** (b0 + (b1/t)) or ln(Y) = b0 + (b1/t). 9) Logistic. Model whose equation is Y = 1 / (1/u + (b0 * (b1**t))) or ln(1/y-1/u) = ln (b0) + (ln(b1) * t) where u is the upper boundary value. 10) Growth. Model whose equation is Y = e** (b0 + (b1 * t)) or ln(Y) = b0 + (b1 * t). 11) Exponential. Model whose equation is Y = b0 * (e**(b1 * t)) or ln(Y) = ln(b0) + (b1 * t). The R Square statistic was used to measure the strength of relationship between the model and the dependent variable. The standard error of the regression was used to compare fits between these types of models. A small standard error of the regression indicates that the data points are closer to the fitted values. After fitting a regression model, the residual plots were checked to be sure that we have unbiased estimates.

## Results

Table 1 shows the number of confirmed COVID-19 cases, recovered cases, and COVID-19 specific deaths during the period 20 February- 21 April, 2020 in 21 Eastern Mediterranean countries. The first confirmed cases in the region were observed in Iran and Egypt on 20 February 2020. During this observed period, the total number of confirmed cases in the region was 138673, the total number of recovered cases was 71343, and the number of deaths from COVID-19 was 6291. The case fatality rate ranged from 0.14% in Qatar to 11.21% in Sudan. The overall fatality rate in the region was 4.5%.

**Table 1:** The number of confirmed COVID-19 cases, recovered cases, and COVID-19 specific deaths during the period 20 February- 21 April, 2020 in Eastern Mediterranean countries

Country	Date of first case	Number of confirmed cases	Number of recovered cases	Number of deaths	Case-fatality rate (%)
Afghanistan	24/2/2020	1092	150	36	3.30
Occupied Palestinian Territory	7/3/2020	329	71	2	0.61
United Arab Emirates	20/2/2020	7755	1443	46	0.59
Jordan	2/3/2020	425	282	7	1.65
Iran	20/2/2020	84802	60965	5297	6.25
Pakistan	27/2/2020	9505	2073	197	2.07
Bahrain	24/2/2020	1952	783	7	0.36
Tunisia	2/3/2020	884	148	38	4.30
Djibouti	18/3/2020	945	112	2	0.21
Sudan	13/3/2020	107	8	12	11.21
Syria	23/3/2020	39	6	3	7.69
Somalia	16/3/2020	237	4	8	3.38
Iraq	24/2/2020	1602	1096	83	5.18
Oman	24/2/2020	1508	238	8	0.53
Qatar	29/2/2020	6533	614	9	0.14
Kuwait	24/2/2020	2080	412	11	0.53
Lebanon	22/2/2020	677	103	21	3.10
Libya	26/3/2020	51	15	1	1.96
Egypt	2/3/2020	3333	821	250	7.50
Morocco	3/3/2020	3186	359	144	4.52
Saudi Arabia	2/3/2020	11631	1640	109	0.94

**Figure 1:**
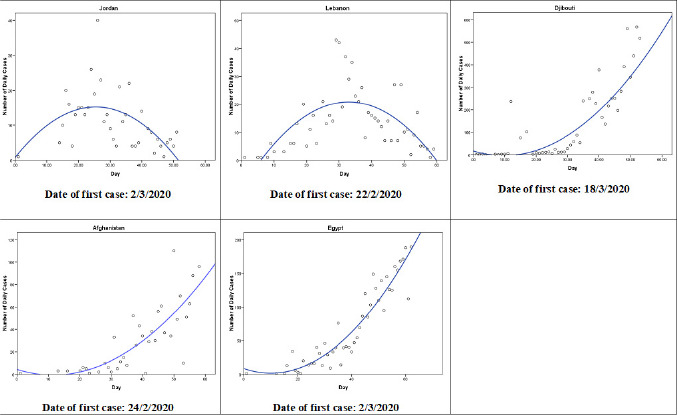
The quadratic models that best fit the observed number of COVID-19 cases until 21<sup>th</sup> April 2020 in Jordan, Lebanon, Djibouti, Afghanistan, and Egypt (Y = b0 + (b1

**Curve fitting:** a scatterplot of the reported COVID-19 cases for each country revealed that the relationship is nonlinear in all countries. Table 2 shows the summary of best fitting model and the parameters estimates for each country. For each country, the model that has high values of R Square and Adjusted R Square and low values of the standard error of the estimates was selected. Other models were tested but they presented average to poor fit, despite that some models were significant. The quadratic model (Y = b0 + (b1 * t) + (b2 * t**2)) follows the observed data points fairly well during the observed time period in five countries: Jordan, Lebanon, Djibouti, Afghanistan, and Egypt (Figure 1). The pattern of the daily confirmed cases in Jordan is similar to that in Lebanon. The quadratic terms were negative for the models in Jordan and Lebanon which clearly indicate that the number of cases increased initially until a turning point was reached. Beyond that value, the number of cases decreased over time. On the other hand, the quadratic terms were negatives for models fitted for the cases in Afghanistan, Djibouti, and Egypt which indicates that the net increase in the number of cases is a greater than linear increase.

**Table 2:** The summary of best fitting model and the parameters estimates for each country

Country	Equation	Model Summary					Parameter Estimates			
		R Square	F	df1	df2	p-value	Constant	b1	b2	b3
Afghanistan	Quadratic	0.65	30.4	2	33	0.000	4.29	-0.86	0.04	
Jordan	Quadratic	0.31	7.9	2	35	0.002	0.73	1.14	-0.02	
Egypt	Quadratic	0.89	178.1	2	45	0.000	8.48	-1.36	0.07	
Lebanon	Quadratic	0.45	19.1	2	47	0.000	-10.28	1.89	-0.03	
Djibouti	Quadratic	0.77	40.2	2	24	0.000	17.40	-4.24	0.22	
Iran	Cubic	0.82	88.3	3	58	0.000	87.84	-24.40	4.58	-0.064
Occupied Palestinian Territory	Cubic	0.22	3.6	3	37	0.023	9.04	-1.46	0.10	-0.002
Sudan	Cubic	0.67	8.2	3	12	0.003	0.38	0.54	-0.05	0.001
Syria	Cubic	0.38	1.4	3	7	0.316	1.94	0.01	0.03	-0.001
Tunisia	Cubic	0.42	8.6	3	36	0.000	-7.94	0.40	0.08	-0.002
Qatar	Cubic	0.82	64.9	3	42	0.000	2.85	4.32	-0.30	0.008
Saudi Arabia	Cubic	0.94	236.1	3	44	0.000	-147.75	36.70	-2.01	0.035
Iraq	Cubic	0.66	29.5	3	46	0.000	11.6	-2.93	0.21	-0.003
Libya	Cubic	0.10	0.5	3	13	0.710	3.60	-0.73	0.08	-0.002
United Arab Emirates	Growth	0.84	191.1	1	37	0.000	0.16	0.11		
Pakistan	Growth	0.74	122.8	1	44	0.000	0.53	0.12		
Bahrain	Growth	0.45	43.6	1	54	0.000	1.20	0.05		
Kuwait	Compound	0.66	100.6	1	52	0.000	1.74	1.08		
Somalia	Compound	0.75	39.2	1	13	0.000	0.27	1.14		
Oman	Exponential	0.85	249.8	1	44	0.000	0.77	0.09		
Morocco	Exponential	0.82	199.2	1	43	0.000	1.20	0.12		

**Figure 2:**
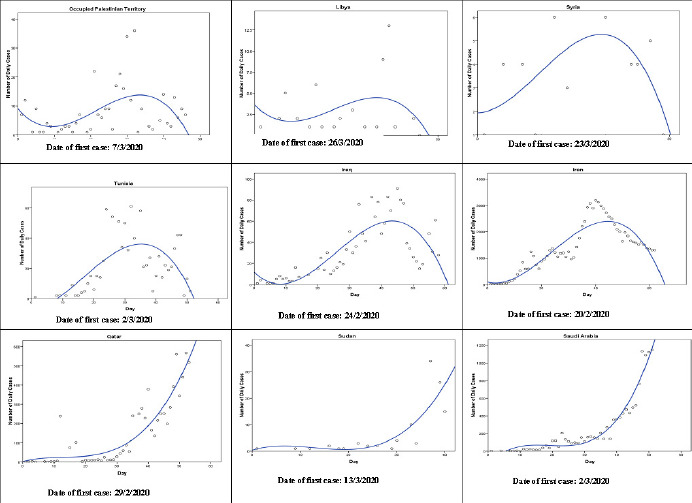
The cubic models that best fit the observed number of COVID-19 cases until 21<sup>th</sup> April 2020 in Occupied Palestinian Territory, Iran, Iraq, Tunisia, Sudan, Syria, Qatar, Libya, and Saudi Arabia (Y = b0 + (b1

**Figure 3:**
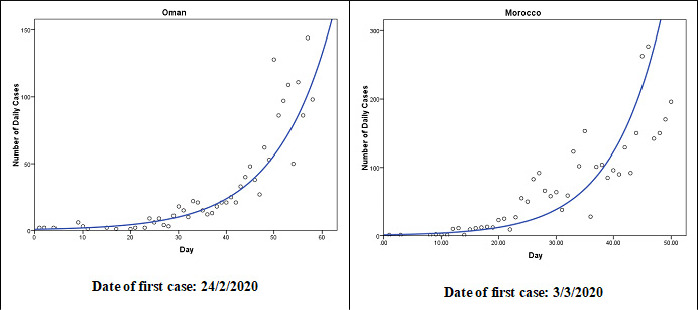
The exponential models that best fit the observed number of COVID-19 cases until 21<sup>th</sup>April 2020 in Oman and Morocco (Y = b0

**Figure 4:**
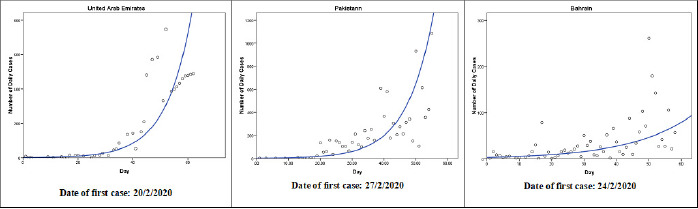
The growth models that best fit the observed number of COVID-19 cases until 21<sup>th</sup> April 2020 in United Arab Emirates, Pakistan, and Bahrain (Y = e

**Figure 5:**
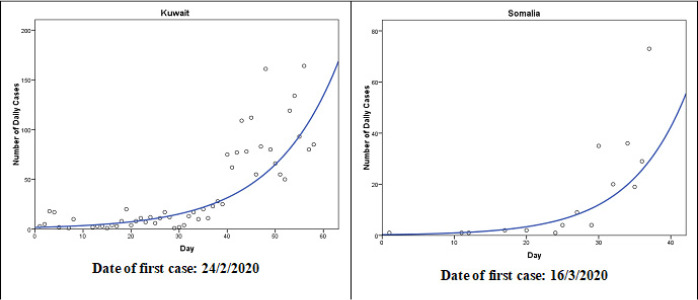
The compound models that best fit the observed number of COVID-19 cases until 21<sup>th</sup> April 2020 in Somalia and Kuwait (Y = b0

The cubic model (Y = b0 + (b1 * t) + (b2 * t**2) + (b3 * t**3)) follows the observed data points fairly well during the observed time period in nine countries: Occupied Palestinian Territory, Iran, Iraq, Tunisia, Sudan, Syria, Qatar, Libya, and Saudi Arabia (Figure 2). The patterns in Occupied Palestinian Territory and Libya were similar. After few initial cases, the number of cases decreased until a turning point and then increased. After another turning point, the number started to decrease. In Qatar, Sudan and Saudi Arabia, the number of cases are still increasing as of the last observed date. The exponential model (Y = b0 * (e**(b1 * t))) follows the observed data points fairly well during the observed time period in two countries: Oman and Morocco (Figure 3). In the two counties, the number of cases grows exponentially, or grows at an increasingly larger rate over time. In United Arab Emirates, Pakistan, and Bahrain, the growth model (Y = e**(b0 + (b1 * t))) was the best fit for the during the observed time period (Figure 4). The compound model (Y = b0 * (b1**t)) was the best fit for the data during the observed time period in Somalia and Kuwait (Figure 5).

## Discussion

The findings of this study are valuable to aid policy makers and healthcare professionals to use the best models and regression equations to predict future cases in their countries. The study demonstrated the best curve-fitting model for each country. To make predictions, these curve-fitting models and regression equations can be used to extrapolate curves into the future. However, curve-fitting models are useful for making very short-term predictions. Looking for how the pandemic will be at longer-term, attention should be paid to transmission models after considering their own built-in uncertainties. The pattern of COVID-19 spread differed between countries in the EMR. However, the interpretation of the inter-country differences in the trends and patterns of COVID-19 spreads is problematic because these countries varied in their capacities of testing and in the implementation of public health measure for prevention and control such as social physical, isolation, sanitation and use of personal protective measure. Therefore, surges in confirmed cases could reflect more testing is being done or actual increase in infections.

Some of the countries of the region have implemented public measures such as rapid case identification, rapid testing and isolation of cases, contact tracing and have introduced widespread physical distancing measures and movement restriction. Consequently, the number of COVID-19 cases in some countries was suppressed below the threshold at which health systems become unable to prevent excess mortality. For these countries, there should be a plan for a phased transition to reduce such restrictions in a manner that will enable the sustainable suppression of transmission at a low-level whilst enabling the resumption of some parts of economic and social life. For other countries where community transmission has led to outbreaks with near exponential growth, widespread population-level physical distancing measures and movement restrictions are needed in order to slow spread and set in place other control measures. For countries that currently have few reported cases such as Yemen, there is a need to explore the situation closer and an opportunity to learn lessons from other countries that succeeded to contain the transmission of the disease. New measures that could provide good or better protection with less social cost are urgently needed [5].

## Conclusion

The pattern of COVID-19 spread differed between countries in the EMR. This might reflect the variations in testing and implementation of public health measure for prevention and control. The best curve-fitting model was demonstrated for each country that can be used for making very short-term predictions.

### What is known about this topic

- Epidemiological models during outbreaks are very helpful for policy makers and public health professionals to make key decisions about the appropriate public health measures and interventions.

### What this study adds

- The trend of the reported COVID-19 cases is nonlinear in all countries in the Eastern Mediterranean Region;
- The number of COVID-19 cases in some countries in the Eastern Mediterranean Region was suppressed below the threshold at which health systems become unable to prevent excess mortality.

## Competing interests

The authors declare no competing interests.
